# RETRACTED: Agrawal et al. *Artemisia* Extracts and Artemisinin-Based Antimalarials for COVID-19 Management: Could These Be Effective Antivirals for COVID-19 Treatment? *Molecules* 2022, *27*, 3828

**DOI:** 10.3390/molecules28217337

**Published:** 2023-10-30

**Authors:** Pawan K. Agrawal, Chandan Agrawal, Gerald Blunden

**Affiliations:** 1 Natural Product Inc., 7963 Anderson Park Lane, Westerville, OH 43081, USA; editorial@naturalproduct.us; 2 School of Pharmacy and Biomedical Science, University of Portsmouth, Portsmouth PO1 2DT, UK; gblunden10@gmail.com

The journal retracts the article “*Artemisia* Extracts and Artemisinin-Based Antimalarials for COVID-19 Management: Could These Be Effective Antivirals for COVID-19 Treatment?” [1]. 

Following publication, concerns were brought to the attention of the Editorial Office regarding significant overlap with a previously published work [2] with a different authorship group and without appropriate citation. 

Adhering to our complaint’s procedure, an investigation was conducted by the Editorial Office and Editorial Board and confirmed the overlap, which included a number of identical sequences of references, abbreviations not commonly used in *Molecules*, as well as the lack of methodology that differentiates the current results from previous publications. This article is therefore retracted.

This retraction was approved by the Editor in Chief of the journal *Molecules*. 

The authors disagree with this retraction. 
